# Supplementary material for: Acute toxicity and responses of antioxidant systems to dibutyl phthalate in neonate and adult Daphnia magna
Source: PeerJ. 2019 Mar 14;7:e6584. doi: 10.7717/peerj.6584 (PMC6421057; doi:10.7717/peerj.6584)
Supplement: Supplemental Information 2 [file peerj-07-6584-s002.docx]

**Supplemental Table S1**

| Nominal(mg/L) | Control/  Vehicle control | 0.50 | 1.00 | 2.00 | 3.00 | 4.00 | 5.00 |
| --- | --- | --- | --- | --- | --- | --- | --- |
| Measured(mg/L**)** | － | 0.5 | 0.90 | 2.0 | 2.65 | 3.58 | 4.00 |
